# MOWDOC: A Dataset of Documents From *Taking the Measure of Work* for Building a Latent Semantic Analysis Space

**DOI:** 10.3389/fpsyg.2020.523494

**Published:** 2021-02-03

**Authors:** Kim F. Nimon

**Affiliations:** Human Resource Development, University of Texas at Tyler, Tyler, TX, United States

**Keywords:** latent semantic analysis, semantic survey response theory, surveys, jingle-jangle fallacies, work, organization

## Introduction

For organizational researchers employing surveys, understanding the semantic link between and among survey items and responses is key. Researchers like Schwarz (1999) have long understood, for example, that item order can impact survey responses. To account for “item wording similarity,” researchers may allow item error variances to correlate (cf. Rich et al., 2010, p. 625). Other researchers, such as Newman et al. (2010), have pointed to semantic similarity between items as support for the premise that work engagement is like old wine in a new bottle.

Recently, organizational researchers (e.g., Arnulf et al., 2014, 2018) have been able to use latent semantic analysis (LSA) and semantic survey response theory (SSRT) to quantify the semantic similarity between and among scales, items, and survey responses. Latent semantic analysis is a computational model that assesses similarity in language where the similarity of any “given word (or series of words) is given by the context where this word is usually found” (Arnulf et al., 2020, p. 4). Latent semantic analysis involves establishing a semantic space from a corpus of existing documents (e.g., journal articles, newspaper stories, item sets). The corpus of documents is represented in a word-by-document matrix and then transformed into an LSA space through singular value decomposition. The reduced LSA space can be used to assess the semantic similarity of documents within the space as well as new documents that are projected onto the space.

Patterns of semantic similarity resulting from LSA have accounted for a substantive amount of variability in how individuals respond to survey items that purport to measure (a) transformational leadership, motivation, and self-reported work outcomes (60–86%; Arnulf et al., 2014), (b) employee engagement and job satisfaction (25–69%; Nimon et al., 2016), and (c) perceptions of a trainee program, intrinsic motivation, and work outcomes (31–55%, Arnulf et al., 2019). It also appears that personality, demographics, professional training, and interest in the subject matter have an impact on the degree to which an individual's responses follow a semantically predictable pattern (Arnulf et al., 2018; Arnulf and Larsen, 2020, Arnulf et al., 2020). While being able to objectively access the degree to which survey responses are impacted by semantics is a great step forward in survey research, such research is often conducted with LSA spaces that are not open and therefore not customizable except by those that have access to the body of text upon which the LSA space is built. In this day of open science, researchers need access not only to the LSA space on which semantic survey research may be based but also to the underlying corpus of text to determine whether choices made in the generation of the LSA space have an impact on the results found.

Researchers may not be able to create their own LSA spaces for a number of reasons, including the fact that on some occasions it is difficult to collect a representative corpus of text (Quesada, 2011). However, building an LSA space allows researchers to customize the space including the application of weighting schemes and the level of dimensionality for the LSA space. As shown by Arnulf et al. (2018), the dimensionality of the LSA space is a factor when using an LSA space to predict empirical correlations from scale item cosines. To help address the barrier to creating an LSA space for use in the analysis of scale items in organizational research, this report provides a dataset of documents from measures reviewed in *Taking the Measure of Work*. In *Taking the Measure of Work*, Fields provided the items for 324 scales and subscales which cover the areas of job satisfaction, organizational commitment, job characteristics, job stress, job roles, organizational justice, work-family conflict, person-organization fit, work behaviors, and work values. The MOWDOC dataset presented in this manuscript provides the documents necessary to create a semantic space from the item sets presented in Fields's *Taking the Measure of Work*.

## MOWDOC

The dataset presented in this manuscript can be accessed via https://doi.org/10.6084/m9.figshare.13298165. The dataset contains five variables for each of the 324 scales and subscales in Fields (2002). The variable *ScaleName* identifies the name of the measure as reported in Fields as well as subscale(s) as appropriate, where subscale names are preceded by a colon. The variable *ScaleRef* identifies the reference from which Fields obtained the items.

The variable *ScaleID* is a unique identifier for each scale/subscale. The first two characters of *ScaleID* identify the type of measure as delineated by Fields (2002), where JS denotes job satisfaction, OC organizational commitment, JC job characteristics, JT job stress, JR job roles, OJ organizational justice, WC work-family conflict, PO person organization fit, WB work behaviors, and WV work values. The next three characters identify the page number on which the item set first appears in Fields. The remaining characters denote subscale(s) as appropriate.

The variable *ScaleDoc* contains the document text for each scale/subscale. The scale documents were created as follows. Item texts and associated metadata from Fields (2002) were manually entered into a comma delimited file and verified by an independent and separate individual. To create the variable *ScaleDoc*, an R script was used to create a character vector by merging all item texts for a given scale/subscale where measures containing multiple item sets or subscales were treated as separate documents. The character vector was tokenized using the *tokens* function from the *quanteda* package (Benoit et al., 2018), which also removed all characters in the Unicode “Punctuation” [P] class. The tokens were then sorted so as to not violate the copyright of the scale publishers. Finally, the tokens were merged into a single character vector.

The variable *ScaleSize* identifies the number of words for each measure that ranges from 3 to 563. The *hedonism* subscale from the Work Values Survey (Schwartz, 1994) has the fewest with two items and the Inventory of Stressful Events (Motowidlo et al., 1986) has the largest with 45 items. The mean number of words across all scales is 67 with an SD of 60. Across all 324 documents, there are a total of 21,741 words.

## Example Usage

The R code that demonstrates how the MOWDOC dataset can be used to create an LSA space and fold a new scale^1^ into the created LSA space can be accessed at https://doi.org/10.6084/m9.figshare.13298177. In general, the code follows the example in Wild (2007) and the Wild (2015) demonstration of the famous Landauer et al. (1998) example. Document-feature matrices were created using the *dfm* function from the *quanteda* package (Benoit et al., 2018), rather than using the *textmatrix* function in the *lsa* package (Wild, 2015). Amongst other differences, the *dfm* function optimally creates a sparse matrix of documents and features.

Here is the R code following a typical LSA process:

First, a text matrix was constructed using the input text. In the demonstration provided, five different document-feature matrices and associated word clouds were created to illustrate the nuances associated with stemming words and removing stop words.

Second, an LSA space with full dimensionality was created and used to verify that the document-feature matrix could be reproduced.

Third, an LSA space with reduced dimensionality was created.

Fourth, document-to-document correlations and cosines were computed using the original document-feature matrix and the reduced LSA space.

Fifth, a new document was folded into the reduced LSA space.

Sixth, correlations and cosines with the new document were created.

## Strengths and Limitations

**Figure 1:**
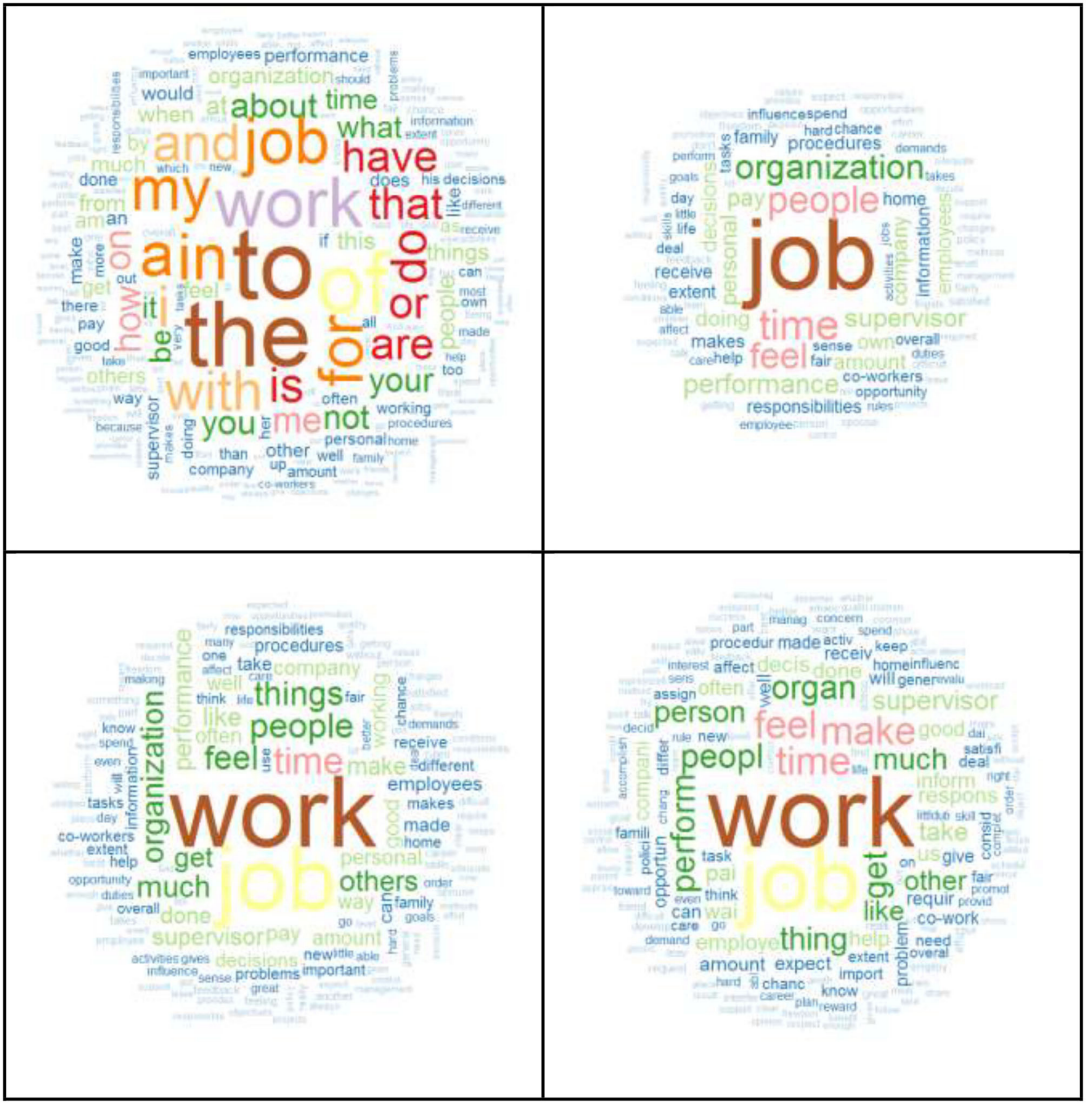
Word clouds on MOWDOC document-feature matrices. Upper-left figure based on matrix with no stemming or stop words removed. Upper-right figure based on matrix with no stemming and English stop words from the *lsa* package (Wild, 2015) removed. Lower-left figure based on matrix with no stemming and English stop words from the *quanteda* package (Benoit et al., 2018) removed. Lower-right figure based on matrix with stemming and English stop words from the *quanteda* package (Benoit et al., 2018) removed.

The MOWDOC datasets contains the item texts for the scales/subscales in the book of *Taking the Measure of Work*. With this dataset, researchers can customize their LSA spaces to fit their research interests including the consideration of stop words, word stemming, and weighting schemes. Note, for example, the differences in the word clouds represented in Figure 1 that result when the MOWDOC dataset was used to generate a document-feature matrix with different parameters. Not only did each document-feature matrix contain a different number of features^2^, the word most frequently used across multiple scales differed according to how the document texts were “sanitized” (cf. Wild, 2007). In the matrix that hasn't been sanitized with no stemming or removal of stop words, “to” occurs in 76% (247) of the scales/subscales. When the matrix has no stemming but English stop words from the *lsa* package (Wild, 2015) are removed, “job” occurred in 48% (157) of the scales/subscales. A matrix with no stemming although English stop words from the *quanteda* package (Benoit et al., 2018) have been removed, “work” occurred in 53% (172) of the scales/subscales. With stemming and English stop words from the *quanteda* package (Benoit et al., 2018) removed, “work” occurred in 56% (181) of the scales/subscales. While it should not come as a surprise that “work” is the predominant word used across scales published in a book that considers the “Measure of Work,” it could be considered problematic to create an LSA space where such a relevant word was removed.

While making the document texts upon which to build an LSA space available is a strength, it might also be a limitation as resulting LSA spaces may yield over-fitted solutions when researchers assess the semantic similarity of item sets (cf. Larsen et al., 2008). It might also be a limitation that the document-feature matrices from the MOWDOC dataset tend to be sparse. Across the different “sanitization” schemes previously outlined, all matrices were at least 98.4% sparse. The dataset is also limited in that it did not preserve the word order of the original item sets. As a reviewer noted, this limits the use of the dataset to document-based models like LSA. In addition, the dataset is small for a source corpus for LSA. With 324 documents and 2,564 unique words, the use of the MOWDOC dataset may be limited beyond the example usage presented.

Clearly more research is needed to determine how the MOWDOC dataset can validly be used to inform survey research. However, even with the stated limitations, the MOWDOC dataset appears to be useful. Take for example the lsaCos.csv file that results from running the demonstration code located at https://doi.org/10.6084/m9.figshare.13298177. It yields the cosines between scales/subscales from the LSA space that was built using a document-feature matrix that was stemmed and void of English stop words contained in the *quanteda* package (Benoit et al., 2018). Notably, the cosine between the *OCBO* item set Williams and Anderson (1991, WB241B) and the *generalized compliance* item set from Smith et al. (1983, WB245B) is 0.92. Interestingly, the cosine reflects the fact that some of the items representing *OCBO*, including “attendance at work is above the norm” and “great deal of time spent with personal phone conversation,” were selected from the Smith et al. (1983) generalized compliance scale.

Researchers might also fold additional items sets onto the LSA space built from *Taking the Measure of Work* to assess their semantic similarity with item sets presented in Fields (2002). For example, folding the Hackman and Oldham (1980) *job satisfaction* item set into the LSA space previously described yields a high cosine (0.86) with the *general satisfaction* item set from Jackman and Oldham (1974). Future work could include adding item texts from other compendiums of organizational research scales including those of Cook et al. (1981), Price and Mueller (1986), and Hersen and Thomas (2003), as well as submitting the existing dataset to the Semantic Scale Network offered by Rosenbusch et al. (2020).

## Author Contributions

The author confirms being the sole contribution of this work and has approved it for publication.

## Conflict of Interest

The author declares that the research was conducted in the absence of any commercial or financial relationships that could be construed as a potential conflict of interest.

## Footnotes

^1^The file JS.csv contains the items for the Hackman and Oldham (1980) job satisfaction scale and can be accessed at https://doi.org/10.6084/m9.figshare.13298168

^2^The document-feature matrix with no stemming or removal of stop words yielded 2,564 features (7.9 features on average per scale) and was 98.5% sparse. The document-feature matrix with no stemming and English stop words from the *lsa* package (Wild, 2015) removed yielded 2,253 features (7.0 features on average per scale) and was 99.2% sparse. The document-term matrix with no stemming and English stop words from the *quanteda* package (Benoit et al., 2018) removed yielded 2,433 features (7.5 features on average per scale) and was 99.0% sparse. The document-term matrix with stemming and English stop words from the *quanteda* package (Benoit et al., 2018) removed yielded 1,704 features (5.3 features on average per scale) and was 98.6% sparse.
